# Return to work after acromioclavicular joint stabilization: a retrospective case control study

**DOI:** 10.1186/s13018-019-1071-7

**Published:** 2019-02-12

**Authors:** Felix Porschke, Marc Schnetzke, Stefan Studier-Fischer, Paul Alfred Gruetzner, Thorsten Guehring

**Affiliations:** BG Trauma Center Ludwigshafen, Heidelberg University Hospital, Ludwig-Guttmann-Straße 13, 67071 Ludwigshafen, Germany

**Keywords:** Acromioclavicular joint, Acromioclavicular stabilization, Tightrope, Endobutton, Return to work, Work

## Abstract

**Background:**

Considering the epidemiology of acromioclavicular (AC) dislocation related to young and active patients, the impact on working capacity is highly relevant. The purpose of this study was to determine the capacity of work and time to return to work (RTW) after AC joint stabilization. We hypothesized that manual working patients show more restrictions returning to work.

**Methods:**

In this retrospective case series, pre- and posttraumatic working capacity of 54 patients (FU-rate 80.1%, FU time 23, range 18–45 month) stabilized in single TightRope technique was analyzed. Clinical outcome (DASH, Constant-Murley score) and complications were evaluated in addition.

**Results:**

Fifty one of 54 patients (94.5%) were returned to work at final follow-up. The median time to return was 13 (5–143) weeks. Manual working patients showed lower RTW-rates (91.2% vs. 100%; *p* = .151), longer RTW-time (15.5 vs. 6 weeks; *p* = .008), and more often persistent shoulder symptoms at work (55.9% vs. 5%; *p* < .001).

**Conclusion:**

After stabilization of AC joint dislocation, the majority of patients returned to work, needing substantial time to return. Manual working patients required more time and often suffer under persistent symptoms at work.

## Background

Dislocations of the acromioclavicular (AC) joint are frequently occurring injuries, particularly in active patients. With an estimated incidence of 1.5–2 per 10,000 inhabitants per year, it is a very common shoulder injury [1–3]. Depending on severity of the injury and the physical demands of the patient, a persistent instability can lead to impairment of shoulder function and physical activity [4]. As AC dislocation frequently occurs in a young and active patient cohort [2, 3, 5], the impact on work capacity is relevant.

A conservative or surgical treatment can be chosen with respect to the degree of dislocation. For the treatment of high-grade dislocations (type IV–VI according Rockwood), multiple surgical techniques have been described in the literature. Here, no technique has been shown to be of gold standard yet. The sole fixation of the AC joint, for example with K-wires, is hardly recommended any more [4]. Non-anatomical techniques such as the Weaver-Dunn or Mumford procedure, in which the lateral clavicle is resected, can lead to a distinct clinical improvement, especially in chronic AC injuries [6, 7].

Anatomic reconstruction of the coracoclavicular ligaments is an increasingly used treatment option [8]. The endobutton technique uses a synthetic non-absorbable suture system between the coracoid and clavicle to restore vertical stability. Excellent clinical and radiographic results have been published for arthroscopic and mini-open techniques [9–13]. For physically active patients in particular, a surgical stabilization is promoted. Conversely, a surgical stabilization of moderate horizontal instabilities remains controversial, even though it is assumed that active patients, in particular, may benefit from surgery [14].

Recently, we were able to show high return to sports rates after AC joint stabilization, but we also found several limitations and factors that influence the postoperative sports activity [15]. In view of these findings, it may be assumed that patients could similarly experience difficulties in returning to work. However, comprehensive data on this topic are not available yet.

Therefore, the purpose of this follow-up study was to determine the work capacity, time to return to work, and factors that influence convalescents after AC joint stabilization. Based on previous findings, we hypothesized that patients who did manual labor would need a longer time to return to work and would experience considerable restrictions.

## Methods

This retrospective case series was performed in accordance with the Declaration of Helsinki under approval of the local ethics committee (Board of Medical Profession of Rhineland-Palatinate in Mainz (No. 837.009.15/9777)). From all patients, informed consent to participate in the study was obtained. From 2011 to 2014, a consecutive series of 79 patients with AC dislocation type III or V according to Rockwood [16] was treated with single TightRope technique in mini-open technique at a level 1 trauma center. Patients underwent surgery for type V injuries in the absence of any contraindications for surgery. For type III injuries, the treatment decision was drawn individually based on a patient’s age and demands. A subgroup of the patients (type V according to Rockwood) has been enrolled in a recent study [17].

Inclusion criteria were AC dislocation type III or V according to Rockwood, acute (within 4 weeks) AC joint stabilization in single TightRope technique, minimum follow-up of 18 months, and written informed consent.

Exclusion criteria were concomitant injury of upper extremity, shoulder problems concerning the injured or contralateral side before injury, and age < 18 or > 65 years.

**Fig. 1:**
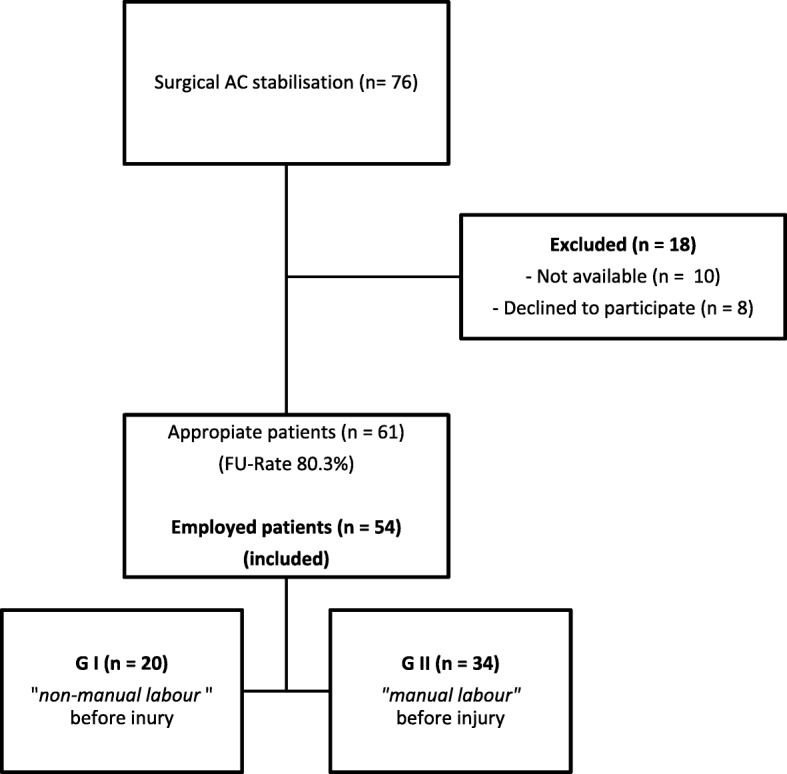
Flow chart; Group distribution

After applying these criteria, 61 patients were identified (follow-up rate 80.3%). Of those, 54 patients were employed at the time of the trauma and were included to this study (Fig. 1).

All 54 patients completed a questionnaire regarding period of convalescence and pre- and postoperative work capacity. In addition, the clinical outcome was evaluated using the Constant-Murley Sscore (CMS) for self-assessment [18, 19] and the disability of the arm, shoulder, and hand (DASH) questionnaire [20]. Complications and revisions were also assessed.

### Surgical procedure and postoperative care

All operations were performed by one of two shoulder specialists (SSF, TG) in a standardized surgical procedure using a mini-incision single TightRope technique initially described by Beris et al. [21]. The postoperative rehabilitation protocol was described in detail recently [17]. Briefly, the operated shoulder was immobilized in a shoulder abduction orthosis (Medi SAK®; Medi, Bayreuth, Germany) at 30° for 4 weeks. Active range of motion on all ranges was allowed after 6 weeks. Three months after surgery, carrying weight and non-contact sports were allowed. After 6 months, there were no further restrictions.

### Data acquisition

For all 54 patients, the electronic patient records were reviewed for surgery-related data (injury-surgery time interval, hospitalization time, revisions).

In addition, all patients completed a questionnaire concerning demographic and work-related issues, such as pre- and post-injury working activity, working impairments, duration of incapacity to work, and frequency and duration of postoperative physiotherapy.

We differentiated the type of employment before trauma as non-manual labor (GI) and manual labor (GII).

As manual labor, all occupations were rated that including tasks like lifting, carrying, pushing or pulling objects with mass of 3 kg or more as defined by ISO 11228-1:2003 (Manual handling).

According to this classification, 20 patients were grouped in GI and 34 patients in GII.

Six patients sustained a work-associated trauma resulting in workers’ compensation status (WC). Basic demographic data of the groups are shown in Table 1. The clinical outcome was measured using the Constant-Murley score (CMS) for self-assessment (pts) [18, 19], the age- and gender-adjusted CMS (%) [22], and the DASH score [20].

**Table 1:** Demographic data

Variable	All (*n* = 54)	Non-manual labor GI (*n* = 20)	Manual labor GII (*n* = 34)	p*
Sex				.481
Male	47 (87)	18 (90)	29 (85.3)	
Female	7 (13)	2 (10)	5 (14.7)	
Age, years	41.5 (20–65)	37.1 (20–65)	42.6 (20–61)	.099
Workers compensation status	6 (11.1)	1 (5)	5 (14.7)	.268
Type V injury	49 (90.7)	18 (90)	31 (91.2)	.619
Concomitant injury	6 (11.1) 1 × vertebral fracture 2 × multiple rip fracture 1 × proximal tibia fracture 1 × contralateral scapula fracture 1 × soft tissue trauma knee	1 (5)	5 (14.7)	.268
Time to surgery, days	10 (1–23)	8 (1–23)	10 (1–22)	.085
Hospitalization time, days	5.0 (1–22)	4.0 (1–11)	5 (2–22)	.028**
Follow-up time, months	23 (18–45)	26 (18–44)	23.5 (18–45)	.529

Continuous data presented as median and minimum/maximum; categorical data as frequencies and percentage

*p value for differences between GI and GII; **p < 0.05, significant

### Statistical analysis

The data was evaluated retrospectively. For continuous variables, mean or median and minimum/maximum values were calculated, and for categorical data, frequencies and percentages are given. Nominal/categorical data were compared using the chi-square test or Wilcoxon signed-rank test. The Mann–Whitney *U* test was used for metric data. The level of significance was defined as *p* = 0.05. A biometrician advised on the statistical analysis. SPSS (version 23.0; SPSS, Chicago, IL) was used for the analysis.

## Results

### Clinical outcome

At final follow-up (23 months; min/max 18–45), the mean-adjusted CMS score for all 54 patients was 89.5% (48.4–100) for the injured shoulder, which was significantly lower than the non-injured contralateral side (96.7% (74.5–100); *p* < 0.001). Manual employees (GII) showed a non-significant trend towards lower clinical outcome compared to non-manual employees (GI) (GI 92.1% (69.2–100) vs. GII 85.2% (48.4–100); *p* = 0.178).

Workers’ compensation status (WC) did not affect the clinical outcome (WC 89.3% (48.4–100) vs. non-WC 87.8% (52.7–100); *p* = 0.645).

Ten patients (18.5%) underwent a revision surgery. There were five early revisions due to wound healing impairments (two with superficial infection) and five late revisions caused by re-instability or hardware-induced soft tissue irritation (Table 2).

**Table 2:** Clinical Outcome

Variable	All (*n* = 54)	Non-manual labor GI (*n* = 20)	Manual labor GII (*n* = 34)	*p* *
Adjusted CMS in %	89.5 (48.4–100)	92.1 (69.2–100)	85.2 (48.4–100)	.178
CMS	84.1 (44.0–100)	87.0 (59.0–100)	79.6 (44–100)	.087
CMS pain	13.5 (4–15)	13.7 (4–15)	13.1 (5–15)	.616
CMS ADL	17.6 (7–20)	18.1 (10–20)	17.1 (7–20)	.095
CMS ROM	34.9 (7–40)	36.7 (24–40)	33.7 (12–40)	.150
CMS strength	18.2 (0–25)	18.5 (6.6–25)	15.8 (4.4–25)	.056
DASH	8.4 (0–68.75)	5.9 (0–35.8)	14.0 (0–68.75)	.056
Work module	12.1 (0–87.5)	0.7 (0–12.5)	13 (0–87.5)	< .001**
Sport module	0 (0–87.5)	11.0 (0–75)	16.8 (0–87.5)	.572
Revisions	10 (18.5)	2 (10)	8 (23.5)	.193
Infections	2 (3.7)	0 (0)	2 (5.9)	.392
Impaired wound healing	3 (5.6)	1 (5)	2 (5.9)	.500
Irritation of hardware	2 (3.7)	0 (0)	2 (5.9)	–
Re-instability	3 (5.6)	0 (0)	3 (8.8)	.241

*CMS* Constant-Murley score, *DASH* Disability of arm, shoulder, and hand

Continuous data presented as mean and minimum/maximum; categorical data as frequencies and percentage

*p value for differences between GI and GII; **p < 0.05, significant

### Convalescence

The median hospitalization time was 5.0 (1–22 days). Manual labor (GI 4.0 (1–11) days vs. GII 5.0, (2–22); *p* = 0.028) was associated with a longer hospitalization time (Table 1). Patients with workers’ compensation status had a longer hospitalization time as well (WC*:* 10.0 SD 2.9 (5–12) days vs. non-WC 4 SD 4.0 (1–22); *p* = 0.006).

**Table 3:** Convalescence and return to work

Variable	All (*n* = 54)	Non-manual labor GI (*n* = 20)	Manual labor GII (*n* = 34)	p*
Return to work	51 (94.4)	20 (100)	31 (91.2)	.151
Temporary incapacity to work	41 (75.9)	11 (55.0)	30 (88.2)	.008*
Time to return to work, w	13.0 (2–143)	6 (2–17)	15.5 (5–143)	< .001**
Persistent shoulder symptoms at work***	18 (34.6)	1 (5)	17 (53.1)	< .001**
Outpatient physiotherapy	29 (53.7)	10 (50%)	19 (55.9)	.445
Duration of outpatient physiotherapy, w	17 (4–147)	18 (7–106)	17 (4–147)	.804

Continuous data presented as mean and minimum/maximum; categorical data as frequencies and percentage

*p value for differences between GI and GII 2; **p < 0.05, significant; *** of the 52 working patients at follow-up

Twenty nine patients (53.7%) performed outpatient physiotherapy. There was no significant difference in the frequency of outpatient physiotherapy between the two groups (GI 50% vs. GII 55.9%; *p* = 0.445).

Outpatient physiotherapy was carried out for a median period of 17 (4–147) weeks, with a frequency of twice (1–6) per week. No significant difference was found between both groups (GI 18.0 vs. GII 17.0 weeks; *p* = 0.804).

Health insurance status did not affect the period of physiotherapy (WC 17.0 (11–118) weeks vs. non-WC 19.0 (4–147) weeks; *p* = 0.978).

### Postoperative working status

**Fig. 2:**
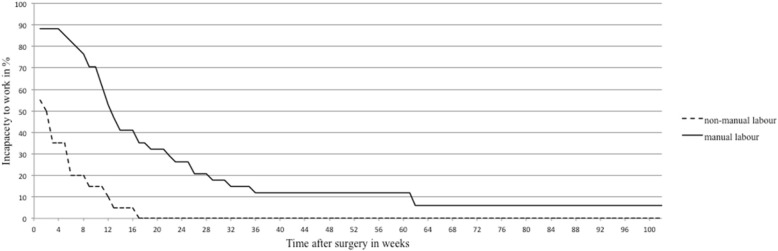
Time of incapacity to work after surgery

A total of 41 patients (75.9%) became temporarily incapacitated to work after surgery (Table 3). Patients who did manual labor reported a temporary incapacity to work significantly more often (GI 55% vs. GII 88.2%; *p* = 0.008) and needed twice as long to return to work compared to non-manual workers (GI 6 vs. GII 15.5 weeks; *p* < 0.001) (Fig. 2).

Patients with workers’ compensation status showed a trend to prolonged incapacity to work (WC 62.0 (11–143) weeks vs. non-WC 12.0 (3–130) weeks; *p* = 0.075).

Of all 54, three patients (5.5%) were unable to return to work during follow-up (two cases of persistent paid sick leave; one case of retraining to do physically less demanding work). All three patients were from the manual labor group (GII), and the not returning to work rate for this group was therefore 8.8%.

At time of follow-up, 18 of the 52 patients (34.6%) who worked complained about persistent symptoms of their operated shoulder. Patients who did manual labor suffered significantly more often from complaints during work (GI 5% vs. GII 53.1%; *p* > 0.001).

## Discussion

The aim of the current study was to determine the rate and time of return to work following acromioclavicular dislocation and surgical stabilization with a flip button system. In particular, we wanted to investigate the relevance of manual labor on the recovery. Our most important finding was that most patients were able to return to work after acromioclavicular stabilization. However, patients who did manual labor were considerably limited in their work capacity. These patients required more than twice as long to return to work and suffered from significantly more persistent pain during working activity. Our hypothesis was therefore confirmed. Furthermore, we found that a relevant proportion of patients who did manual labor (8.8%) did not return to their previous job after a follow-up of 2 years.

The findings also agree with our previous study that reported worse return to sports rates, especially for more physically demanding type of sports [17]. In view of the current data, the notion that physically active patients in particular could benefit from a surgical stabilization of unstable AC joint dislocations appears to be questionable.

Return to work remains an important part of patient satisfaction after surgery and also has economic consequences, especially as AC injury is commonly found in a young and active patient cohort [2, 3, 5]. It is therefore surprising that no comprehensive analyses of work capacity after stabilization have been performed.

AC joint stabilization in endobutton technique is well observed, and mid-term follow-up has shown excellent clinical and radiographic findings [9–12]. However, a well-substantiated statement about work capacity is not available yet.

In a prospective randomized study comparing different endobutton techniques for AC stabilization, Lu et al. report “returning to work” within 6 months for all 80 patients, which is noticeably faster compared to our findings. Detailed information about quality of work or restrictions after surgery is lacking [23].

For delayed AC joint stabilization, autogenous tendons are often used for reconstruction [24]. Recently, Garofalo et al. published a study about simultaneous stabilization of CC and AC ligaments with semitendinosus autograft and this showed good clinical results. A return to work rate of 93% was given here, which is comparable to our findings, but detailed analyses are lacking here as well [25].

Loriaut et al. were the only ones who compared patients with “light” and “heavy” work after endobutton stabilization, and they found a significantly longer time to return to work for patients who did heavy labor (11 vs. 20 weeks) which is comparable to our results [26].

A comparison between patients who do manual labor and those who do not is very rarely found in the literature even for other surgical shoulder procedures. Luyckx et al. found a significantly longer sick leave for patients who do manual labor after subacromial decompression compared to other employees [27]. As might be expected, the duration of return to work after shoulder arthroplasty was higher than in our study. In limitation, it must be mentioned that the authors do not gave an exact definition of manual labor. In our study, we used the ISO definition (ISO 11228-1:2003) of manual handling which is focused on tasks that involve manual handling of objects. It should be considered that other classifications of working activity may have led to different results.

Hurwir et al. recently published data about humeral hemiarthroplasty and reverse total shoulder arthroplasty. Here, only 70% and 65%, respectively, returned to work. Interestingly, they found no difference of return to work with regard to intensity of work performed [28].

For the DASH working subscale, we found significant differences between patients who did manual labor and those who did not. In fact, the subscale was the only clinical score that showed significant differences between both groups in our study. This emphasizes the relevance of a detailed survey of postoperative outcome and might indicate that the important factor of work capacity is underrepresented on the global CMS and DASH scores.

Another focus of our study was the postoperative convalescence course. With 5 days of postoperative hospitalization, the patients in this series showed a relatively long hospital stay compared to other studies. This might in part be due to a specifically German aspect of the health insurance system. Six patients (11.1%) in our investigation were covered by the German Social Accident Insurance. This is similar to workers’ compensation status and permits a more comprehensive rehabilitation concept that involves a prolonged early postoperative in-patient physiotherapy. In fact, these patients had a significantly longer postoperative hospitalization time (10 days vs. 4 days) compared to patients with standard health insurance. The health insurance status did not affect the duration of physiotherapy and the clinical outcome. Our data showed a non-significant trend to longer sick leave for patients with workers’ compensation status.

It came as a surprise that the postoperative physiotherapy was performed by only half of all patients. Every patient received recommendations with detailed protocols regarding postoperative physiotherapy. Ambulant physiotherapy was performed for a median period of 4 months. Patients who did manual labor showed a trend towards prolonged physiotherapy. Interestingly, neither clinical outcome nor the return to work data was associated with presence or absence of postoperative physiotherapy.

In this study, there was no difference concerning clinical outcome and work capacity between moderate- (Rockwood III) and high- (Rockwood V) grade AC dislocation. However, it should be noted that only a few Rockwood type III injuries were included.

In this context, the ongoing debate about the management of type III injuries should be considered. A conservative treatment with immobilization of the injured shoulder for short-term pain reduction only might provide a shorter rehabilitation period and fewer complications. A rare study about conservative treatment of type V instability revealed a return to work rate of only 77% with limited functional outcome score [29]. For younger and physically active patients, in particular, an operative stabilization is often recommended. It is assumed that they benefit from the restoration of the anatomy. Our findings qualify this approach. In our study, patients who did manual labor complained most about persistent symptoms.

The surgical revision rates in this study (five early and five late revisions) were quite high compared to published data [9–11]. All five early revisions were probably due to a prominent knot of the clavicular TightRope suture causing a mechanical irritation. By submerging the suture beneath the deltoid muscle, the early revision rates seemed to decrease. However, there is no statistical evidence for this. The rate of late revisions, including recurrent instability and hardware tenderness, was comparable to existing literature.

The findings of this study are limited by its retrospective design. A mean follow-up time of 23 months was too short to explore the long-term effects of surgical stabilization on work capacity. Due to the limited number of patients included, we pooled all patients who did manual labor even though there are substantial differences between various professions regarding strain on the shoulder. For example, it might have been interesting to investigate the relevance of overhead work.

Due to its retrospective design, there were considerable discrepancies regarding group size and age (37 vs. 42 years) of both groups. In addition in GII group, we found more concomitant injuries (5% vs. 14.7%) without significance. Due to the origin purpose of the study and the small number of concomitant injuries, a multivariate analysis was not conducted. In fact, all three patients that did not return to work had an AC joint dislocation in singularity. But of course, additional injury of the proximal tibia or vertebral fracture might have impact on clinical outcome and return to work.

The fact that 54 patients were operated on by one of two specialized surgeons provides a high standardization of the surgical procedure but could also imply a performance bias. A strength of this study, besides the standardized surgical and rehabilitation protocol, was the large number of patients included compared to previous published studies.

## Conclusions

Most patients who undergo surgical acromioclavicular stabilization using the endobutton technique return to work within 6 months. Patients who did manual labor took more than twice as long as this and suffered from persistent symptoms significantly more often. Furthermore, 8.8% of patients who did manual labor did not return to work within 2 years. In view of these findings, physically active patients need comprehensive information about postoperative work-related restrictions.

## Abbreviations

AC: Acromioclavicular
CMS: Constant-Murley score
DASH: Disability of the arm, shoulder, and hand
WC: Workers’ compensation
